# Supplementary material for: Validation of the rabbit pain behaviour scale (RPBS) to assess acute postoperative pain in rabbits (Oryctolagus cuniculus)
Source: PLoS One. 2022 May 26;17(5):e0268973. doi: 10.1371/journal.pone.0268973 (PMC9135295; doi:10.1371/journal.pone.0268973)
Supplement: S1 Table — (DOCX) [file pone.0268973.s001.docx]

| **RABBIT BEHAVIOUR PAIN SCALE (RPBS)** | |
| --- | --- |
| **Item** | **Score** |
| **1) Posture** | |
| A) Moves around normally and/or jumps | |
| B) Exhibits bipedal or quadrupedal position (with the four limbs extended vertically) | |
| C) Walks at a very slow pace | |
| D) Lies for most of the time | |
| E) Does not move for most of the time | |
| Presence of state A and/or B only* | 0 |
| Presence of one of states C, D, or E | 1 |
| Presence of two or more of states C, D, or E | 2 |
| *The score will only be 0 when there are no C, D or E behaviours | |
| **2) Activity** | |
| A) The rabbit moves normally and/or when stationary performs normal activity* | |
| B) The rabbit moves little and does not perform normal activity | |
| C) The rabbit is immobile and does not perform normal activity | |
| *Interacts with environmental enrichment objects (pine cone, toy and others), eats, drinks water, digs in shavings, exhibits self-cleaning behaviour, sniffs the environment | |
| Presence of state A | 0 |
| Presence of state B | 1 |
| Presence of state C | 2 |
| **3) Interaction and Appetite** | |
| A) Interacts with environmental enrichment objects* | |
| B) Eats** | |
| C) Sniffs the environment | |
| D) Exhibits self-cleaning behaviour (grooming), with the exception of the affected area | |
| The rabbit presents more than one of these behaviours | 0 |
| The rabbit presents one of these behaviours | 1 |
| The rabbit does not present any of these behaviours | 2 |
| *Pine cone, toy, pen substrate |  |
| **Food, vegetables, greens, or snacks. |  |
| **4) Facial Expression** | |
| A) Keeps eyes wide open and ears erect all the time | |
| B) Keeps eyes semi-closed or closed at any moment* | |
| C) Presents drooping ears at any moment | |
| The rabbit displays expression A | 0 |
| The rabbit displays expression B or C | 1 |
| The rabbit displays expressions B and C | 2 |
| *Blinking of the eyes is not considered as semi-closed or closed eyes |  |
| **5) Attention to the affected area** | |
| A) Licks affected area | |
| B) Presses abdomen against the floor | |
| C) Keeps one limb suspended | |
| The rabbit does not present any of these behaviours | 0 |
| The rabbit presents one of these behaviours | 1 |
| The rabbit presents more than one of these behaviours | 2 |
| **6) Miscellaneous behaviours** | |
| A) Attempts to stand up, but remains lying down | |
| B) Has spasms of the skin on the back (twitch) | |
| C) Rapid dorsal movement of the body (flinch) | |
| D) Retracts and closes eyes (wince) | |
| E) Tremors* | |
| The rabbit does not present any of these behaviours | 0 |
| The rabbit presents one of these behaviours | 1 |
| The rabbit presents more than one of these behaviours | 2 |
| *More easily observed in the head and ears |  |
