# Supplementary material for: Validation of the rabbit pain behaviour scale (RPBS) to assess acute postoperative pain in rabbits (Oryctolagus cuniculus)
Source: PLoS One. 2022 May 26;17(5):e0268973. doi: 10.1371/journal.pone.0268973 (PMC9135295; doi:10.1371/journal.pone.0268973)
Supplement: S2 Table — Items with a total score > 0.5 were included in the scale. (DOCX) [file pone.0268973.s002.docx]

| **Expert committee**  **Behaviour (subitem)** | **Animal pain specialist** | **Experienced veterinarian anesthesiologist** | **Laboratory animal veterinarian** | **Item score** |
| --- | --- | --- | --- | --- |
| Moves normal/jumps | 1 | 1 | 1 | **1** |
| Bipedal or quadrupedal | 1 | 1 | 1 | **1** |
| Walks at a very slow pace | 1 | 1 | 0 | **0,7** |
| Lies down | 1 | 1 | 1 | **1** |
| Does not move for most of the time | 1 | 1 | 1 | **1** |
| Normal activity | 1 | 1 | 1 | **1** |
| Moves little | 1 | 1 | 1 | **1** |
| Is immobile | 1 | 1 | 1 | **1** |
| Interacts | 1 | 1 | 0 | **0,7** |
| Eats | 1 | 1 | 1 | **1** |
| Sniffs | 1 | 1 | 1 | **1** |
| Grooms | 1 | 1 | 1 | **1** |
| Eyes open and ears erect | 1 | 1 | 1 | **1** |
| Eyes closed or semi-closed | 1 | 1 | 1 | **1** |
| Ears flat | 1 | 1 | 1 | **1** |
| Licks affected area | 1 | 1 | 1 | **1** |
| Presses abdomen | 1 | 1 | 1 | **1** |
| Limb suspended | 1 | 1 | 1 | **1** |
| Attempts to stand | 1 | 1 | 0 | **0,7** |
| Spasms | 1 | 1 | 1 | **1** |
| Dorsal movement | 1 | 1 | 1 | **1** |
| Retracts and closes eyes | 1 | 1 | 1 | **1** |
| Tremors | 1 | 1 | 1 | **1** |
