# Supplementary material for: Validation of the rabbit pain behaviour scale (RPBS) to assess acute postoperative pain in rabbits (Oryctolagus cuniculus)
Source: PLoS One. 2022 May 26;17(5):e0268973. doi: 10.1371/journal.pone.0268973 (PMC9135295; doi:10.1371/journal.pone.0268973)
Supplement: S3 Table — Adapted from [24]. RPBS: Rabbit pain behaviour scale. Statistical tests according to Table 3: CV—content validation; % pain ≥ 15—at least 15% frequency of occurrence of items/subitems at pain time point); PCA—Principal component analysis (loading value ≥ 0.50 or ≤ -0.50); Intra–intraobserver reliability (> 0.50); Inter–inter-observer reliability (> 0.50); Resp (responsiveness)—higher score of the behaviour at pain time point vs baseline according to Friedman test; ITC—item-total Spearman correlation between 0.3–0.7; IC—Internal consistency (> 0.6); Sp–Specificity (≥ 70%); S–Sensitivity (≥ 70%). The main items were subjected to ten tests and when approved at least in seven, they were included in the final scale (Table 4); the subitems were subjected to seven tests marked with asterisk (*) and when approved in at least three, they were included in the final scale (Table 4). Number 1 indicates that the item/subitem was approved according to the criteria of each test. The items and subitems included in the final scale after refinement are in bold. (DOCX) [file pone.0268973.s003.docx]

| **Items/ Test** | **CV*** | **% pain ≥ 15*** | **PCA** | **Intra*** | **Inter*** | **Resp** | **ITC** | **IC** | **S*** | **Sp*** | **Sum** |
| --- | --- | --- | --- | --- | --- | --- | --- | --- | --- | --- | --- |
| **Posture** | 1 | 1 | 1 | 1 | 1 | 1 | 0 | 1 | 1 | 0 | **8** |
| **Moves normal/jumps** | 1 | 1 |  | 1 | 1 | 1 |  |  | 1 | 0 | **6** |
| **Bipedal or quadrupedal** | 1 | 1 |  | 1 | 1 | 1 |  |  | 1 | 0 | **6** |
| **Walks at a very slow pace** | 1 | 1 |  | 1 | 1 | 0 |  |  | 0 | 1 | **5** |
| **Lies down** | 1 | 1 |  | 1 | 1 | 1 |  |  | 0 | 1 | **6** |
| **Does not move for most of the time** | 1 | 1 |  | 1 | 1 | 1 |  |  | 1 | 0 | **6** |
| **Activity** | 1 | 1 | 1 | 1 | 1 | 1 | 1 | 1 | 0 | 1 | **9** |
| **Interaction and apptetite** | 1 | 1 | 1 | 1 | 1 | 1 | 0 | 1 | 1 | 1 | **9** |
| **Interacts** | 1 | 1 |  | 1 | 1 | 1 |  |  | 1 | 0 | **6** |
| **Eats** | 1 | 1 |  | 1 | 1 | 1 |  |  | 1 | 0 | **6** |
| **Sniffs** | 1 | 1 |  | 1 | 1 | 1 |  |  | 1 | 1 | **7** |
| **Grooms** | 1 | 1 |  | 1 | 1 | 1 |  |  | 0 | 0 | **5** |
| **Facial expression** | 1 | 1 | 1 | 1 | 1 | 1 | 1 | 1 | 1 | 0 | **9** |
| **Eyes open and ears erect** | 1 | 1 |  | 1 | 1 | 1 |  |  | 1 | 0 | **6** |
| **Eyes closed or semi-closed** | 1 | 1 |  | 1 | 1 | 1 |  |  | 1 | 0 | **6** |
| **Ears flat** | 1 | 1 |  | 1 | 1 | 1 |  |  | 0 | 0 | **5** |
| **Attention to the affected area** | 1 | 1 | 0 | 1 | 1 | 1 | 0 | 1 | 1 | 1 | **8** |
| **Licks affected area** | 1 | 1 |  | 1 | 1 | 1 |  |  | 0 | 1 | **6** |
| **Presses abdomen** | 1 | 1 |  | 1 | 1 | 1 |  |  | 0 | 1 | **6** |
| **Limb suspended** | 1 | 1 |  | 1 | 1 | 1 |  |  | 0 | 1 | **6** |
| **Miscellaneous behaviours** | 1 | 1 | 1 | 1 | 1 | 1 | 1 | 1 | 0 | 1 | **9** |
| **Attempts to stand** | 1 | 1 |  | 1 | 1 | 1 |  |  | 0 | 1 | **6** |
| Spasms | 1 | 0 |  | 0 | 0 | 0 |  |  | 0 | 1 | 2 |
| **Dorsal movement** | 1 | 1 |  | 1 | 0 | 1 |  |  | 0 | 1 | **5** |
| **Retracts and close eyes** | 1 | 1 |  | 0 | 0 | 0 |  |  | 0 | 1 | **3** |
| **Tremors** | 1 | 1 |  | 1 | 1 | 1 |  |  | 0 | 1 | **6** |
