# Supplementary material for: Validation of the rabbit pain behaviour scale (RPBS) to assess acute postoperative pain in rabbits (Oryctolagus cuniculus)
Source: PLoS One. 2022 May 26;17(5):e0268973. doi: 10.1371/journal.pone.0268973 (PMC9135295; doi:10.1371/journal.pone.0268973)
Supplement: S4 Table — Interpretation of the degree of reliability kw: very good: 0.81–1.0; good: 0.61–0.80; moderate: 0.41–0.60; reasonable: 0.21–0.4; poor <0.2 [45]. Bold type corresponds to values > 0.61. (DOCX) [file pone.0268973.s004.docx]

| **Items/Evaluators** | **1 x 2** | **1 x 3** | **1 X 4** | **2 X 3** | **2 X 4** | **3 X 4** |
| --- | --- | --- | --- | --- | --- | --- |
| Posture | **0.81 (0.76-0.85)** | **0.83 (0.80-0.87)** | **0.87 (0.84-0.90)** | **0.87 (0.83-0.91)** | **0.87 (0.83-0.91)** | **0.87 (0.84-0.90)** |
| Activity | **0.70 (0.74-0.77)** | **0.86 (0.86-0.86)** | **0.85 (0.85-0.85)** | **0.79 (0.76-0.83)** | **0.79 (0.76-0.83)** | **0.85 (0.85-0.85)** |
| Interaction and appetite | **0.92 (0.92-0.92)** | **0.91 (0.91-0.92)** | **0.92 (0.92-0.92)** | **0.96 (0.96-0.96)** | **0.96 (0.96-0.96)** | **0.92 (0.90-0.93)** |
| Facial Expression | **0.64 (0.58-0.71)** | **0.72 (0.67-0.78)** | **0.79 (0.74-0.84)** | **0.71 (0.65-0.78)** | **0.71 (0.65-0.78)** | **0.73 (0.68-0.78)** |
| Attention to the affected area | **0.74 (0.71-0.77)** | **0.70 (0.64-0.75)** | **0.83 (0.80-0.85)** | **0.79 (0.79-0.79)** | **0.79 (0.79-0.79)** | **0.79 (0.76-0.82)** |
| Miscellaneous behaviours | 0.47 (0.36-0.59) | 0.32(0.21-0.43) | 0.46 (0.37-0.56) | 0.55 (0.48-0.52) | 0.60 (0.53-0.66) | **0.68 (0.64-0.73)** |
